# Microbial Primer: Multidrug efflux pumps

**DOI:** 10.1099/mic.0.001370

**Published:** 2023-10-03

**Authors:** Pauline Ann Siasat, Jessica M. A. Blair

**Affiliations:** ^1^​ College of Medical and Dental Sciences, Institute of Microbiology and Infection, University of Birmingham, B15 2TT Birmingham, UK

**Keywords:** antibiotic resistance, multidrug efflux pumps, RND

## Abstract

Multidrug efflux pumps are molecular machines that sit in the bacterial cell membrane and pump molecules out from either the periplasm or cytoplasm to outside the cell. While involved in a variety of biological roles, they are primarily known for their contribution to antibiotic resistance by limiting the intracellular accumulation of antimicrobial compounds within bacteria. These transporters are often overexpressed in clinical isolates, leading to multidrug-resistant phenotypes. Efflux pumps are classified into several families based on their structure and understanding the characteristics of each family is important for the development of novel therapies to restore antibiotic potency.

## Introduction

Antibiotics are one of the foundations of modern medicine. For most antibiotics to be effective, a sufficient amount of these drugs must enter and accumulate within bacterial cells. To prevent this, bacteria have evolved several resistance mechanisms to combat antibiotic activity, including multidrug efflux pumps.

**Fig. 1.**
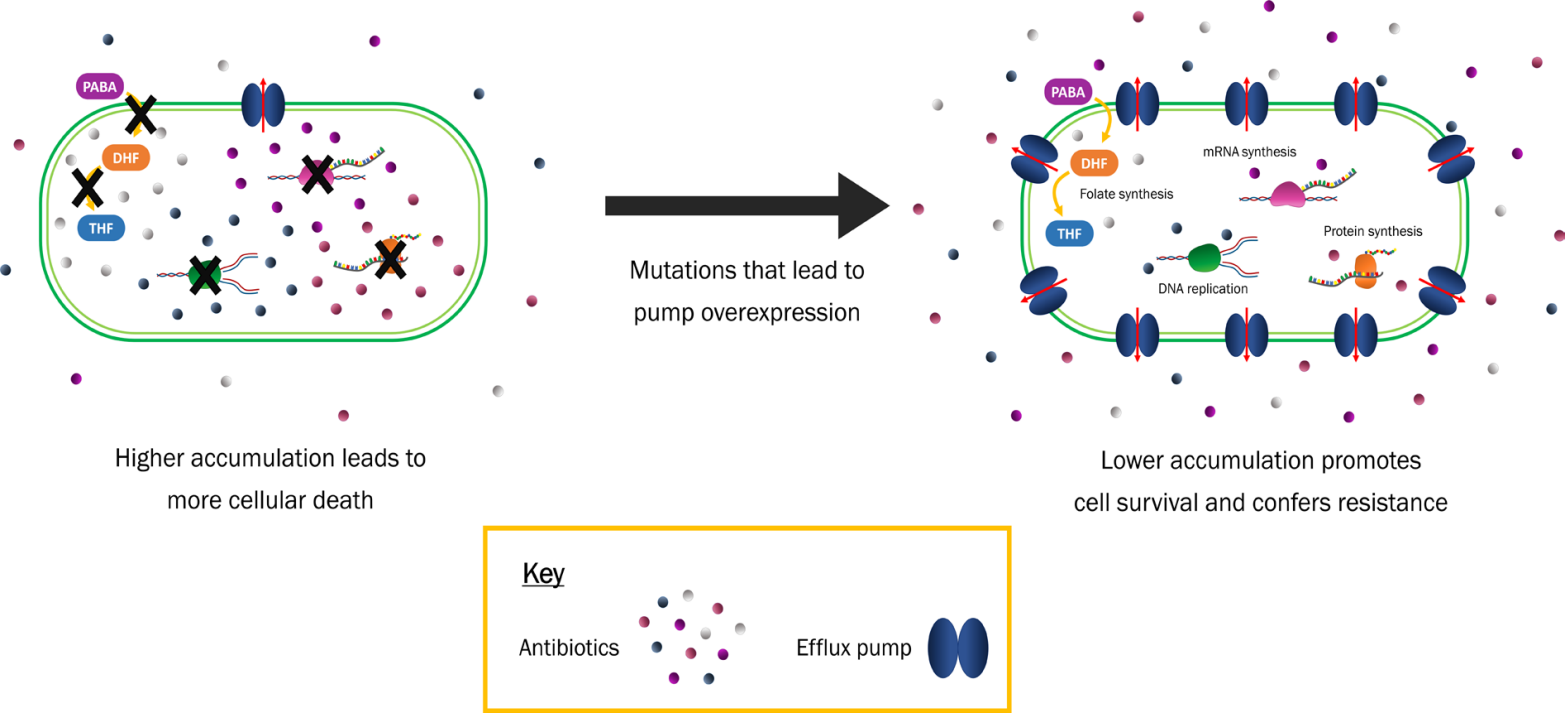
Overexpression of efflux pumps leads to reduced antibiotic accumulation and promotes resistance. The accumulation of antibiotics within bacterial cells prevents essential cellular processes (i.e. DNA replication) from functioning. In this state, bacterial infections can still be managed with existing antimicrobials. However, clinical isolates typically acquire mutations that result in pump overexpression. Consequently, reduced quantities of drugs accumulate inside the cell and bacteria can survive at higher antibiotic concentrations.

Multidrug efflux pumps are protein machines that export substrates from inside cells to the external environment. Following their discovery 30 years ago, these transporters have been established as key drivers of antimicrobial resistance (AMR) because they can pump antibiotic molecules out of bacterial cells (Fig. 1). They serve as the first line of defence against antimicrobials, working in conjunction with the cell membrane of Gram-positive, and the cell envelope of Gram-negative, bacteria. By utilizing energy obtained from either ATP or an electrochemical gradient, these proteins export an extensive range of toxic compounds – including antibiotics, dyes, metals and detergents. While many of these substrates are captured from within the cytoplasm and transported across the inner membrane, in Gram-negative bacteria there are also pumps that remove substrates from the periplasm and export them across the outer membrane.

Efflux pumps contribute to antibiotic resistance by effectively preventing the accumulation of antibiotics inside bacterial cells. Resistance in clinical isolates is further driven by pump overexpression – often resulting in multidrug-resistant (MDR) phenotypes. By providing an intrinsic basal level of resistance to many antibiotics, the overexpression of efflux pumps serves as a platform for the development of other resistance mechanisms.

**Fig. 2.**
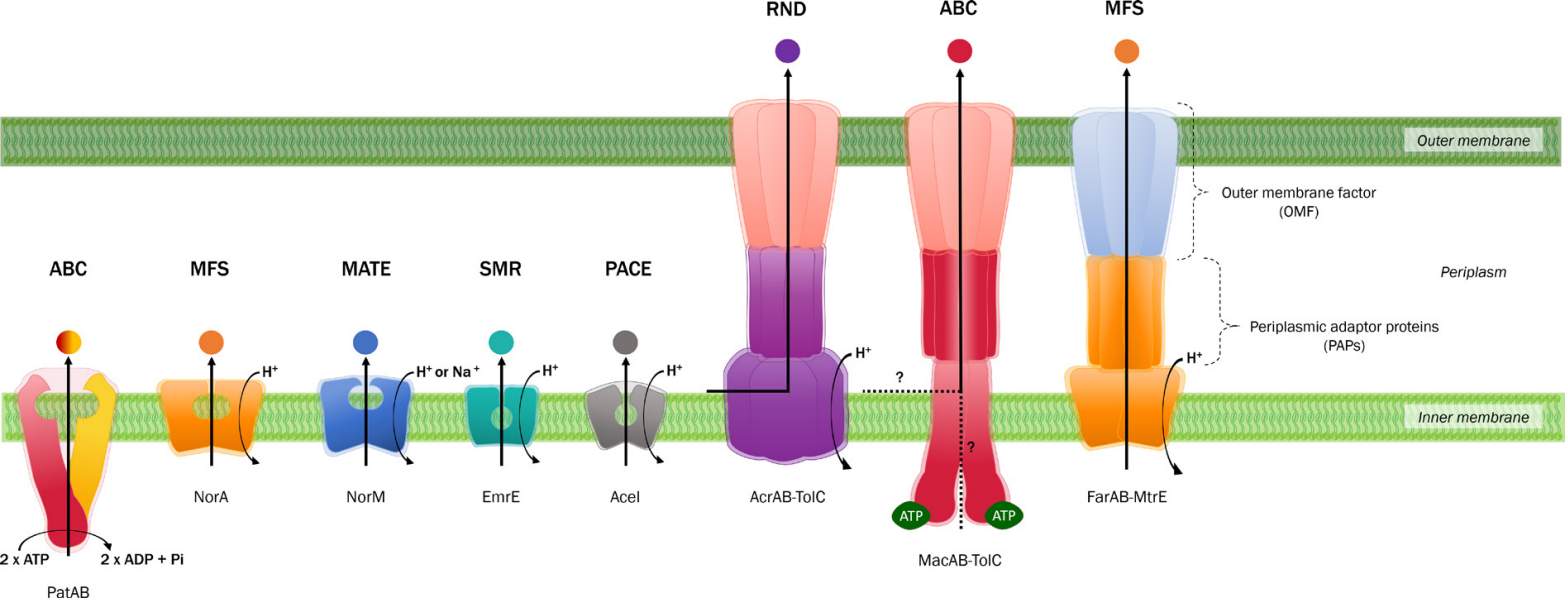
Schematic representations of multidrug efflux pumps and tripartite structures. Efflux pumps are classified into six families: (**i**) ATP-binding cassette (ABC); (ii) major facilitator superfamily (MFS); (iii) multidrug and toxin extrusion (MATE); (iv) small multidrug resistance (SMR); (**v**) proteobacterial antimicrobial compound efflux (PACE); and (vi) resistance–nodulation–cell division (RND). The ABC family utilize ATP as an energy source for substrate efflux; in comparison, the remaining families rely on an electrochemical gradient – primarily proton (H^+^) ions, but MATE pumps can also use sodium ions (Na^+^). RND transporters associate with periplasmic adaptor proteins (PAPs) and an outer membrane factor (OMF) to form tripartite structures. This is also the case with some ABC (i.e. MacAB–TolC) and MFS (i.e. FarAB–MtrE) systems. While ABC pumps canonically transport substrates across the inner membrane, it is hypothesized that MacB captures substrates either from the periplasm or the outer leaflet of the inner membrane – similar to RND pumps. However, further research is required to understand the molecular mechanisms of substrate translocation by ABC-type tripartite efflux systems.

Found in all domains of life, efflux pumps are classified into six families: (i) ATP-binding cassette (ABC); (ii) major facilitator superfamily (MFS); (iii) multidrug and toxin extrusion (MATE); (iv) small multidrug resistance (SMR); (v) proteobacterial antimicrobial compound efflux (PACE); and (vi) resistance–nodulation–cell division (RND) (Fig. 2). Although extensively studied for their role in antibiotic resistance, these transporters are also involved in a variety of physiological functions, including virulence, biofilm formation and cellular metabolism. A combination of efflux pumps from each family are expressed in all species of bacteria, working synergistically to promote fitness and pathogenicity.

## ABC transporters

Members of the ABC family are membrane-bound transporters that utilize ATP directly to power substrate transport. They are functionally diverse, acting as either importers, exporters, or non-transport proteins. In eukaryotic cells, ABC transporters are important contributors to chemoresistance and have been shown to a play role in the multidrug resistance of various cancer cells lines, such as ABCC1 in breast, prostate, renal and thyroid cancer. Moreover, the efflux activity of the ABC family also drives resistance to antifungal medicines, with the overexpression of CDR1p in *Candida auris* and Afr1 in *Cryptococcus neoformans* resulting in diminished susceptibility against fluconazole, a commonly administered antifungal.

ABC transporters typically consist of four domains – two transmembrane domains (TMDs) involved in substrate capture, and two highly conserved nucleotide-binding domains (NBDs) required for ATP hydrolysis. Variations within the TMD architecture contribute to the functional diversity of this family. Bacterial ABC pumps are composed of two polypeptide chains that are either the same (homodimer) or different (heterodimer). Members of the latter group are important mediators of resistance in Gram-positive bacteria. For instance, PatAB efflux pumps have been shown to confer resistance against fluoroquinolones, including ciprofloxacin and norfloxacin, in *Streptococcus pneumoniae *. Likewise, EfrAB of *Enterococcus faecalis* contributes to biocide tolerance and exports gentamicin, streptomycin and chloramphenicol. In comparison, homodimer MacB forms tripartite structures in Gram-negative bacteria, including pathogenic species such as *Escherichia coli *, *Acinetobacter baumannii* and *Neisseria gonorrhoeae *. In association with the outer membrane factor (OMF) TolC and periplasmic adaptor protein (PAP) MacA, MacB facilitates the export of macrolide antibiotics, lipopeptides and virulence factor enterotoxin II.

## MFS transporters

As the largest and most diverse group of membrane transporters, members of the MFS superfamily function as either uniporters (substrate transport without ions), symporters (ion and substrate transport in the same direction), or antiporters (ion and substrate transport in opposite directions). Comprising approximately 400–600 amino acids, MFS transporters share a common architecture of 12–14 transmembrane helices organized into 2 domains attached together by a long and flexible intracellular loop.

Both symporters and antiporters utilize the proton motive force (PMF) to drive transmembrane transport. In bacteria, symporter substrates are typically nutrients required for normal cellular functions, such as sugars and amino acids. In contrast, antiporters function in response to cellular challenge and can confer clinically relevant levels of resistance, particularly in Gram-positive bacteria. A prime example is NorA of *Staphylococcus aureus *, which captures an array of antimicrobial compounds – particularly fluoroquinolones and quaternary ammonium compounds (QACs) such as benzylkonium chloride, a biocide widely used as an antiseptic and disinfectant. In addition, NorA is involved with the formation of biofilms, an important lifestyle that contributes to persistent and chronic nosocomial infections by methicillin-resistant *S. aureus* (MRSA). Another notable MFS pump is P55 of *Mycobacterium tuberculosis *, which confers resistance to aminoglycosides, tetracycline, clofazimine and rifampicin. It also contributes to pathogenicity through its involvement in the uptake of cholesterol, utilized for mycobacterial growth *in vivo* and within macrophages.

While most function as a single component, some MFS transporters form tripartite structures in Gram-negative bacteria. For instance, the FarAB–MtrE system enhances the survival of *N. gonorrhoeae* during infection by extruding long-chained antibacterial fatty acids, such as linoleic acid and palmitic acid. Other structures include VceABC of *Vibrio cholerae *, which exports bile salts, nalidixic acid and chloramphenicol, as well as EmrAB-TolC of *E. coli *, which can extrude mammalian steroid hormones (i.e. estradiol and progresterone) and nalidixic acid.

## MATE transporters

Using energy from transmembrane H^+^ and/or Na^+^ electrochemical gradients across bacterial membranes, MATE transporters remove cationic compounds such as norfloxacin and ethidium bromide. Arranged differently from those in the MFS superfamily, MATE efflux pumps consist of 12 transmembrane helices bundled into 2 domains. While MATE pumps commonly use one or the other, NorM of *V. cholerae* can utilize both ion gradients for substrate extrusion, reportedly due to conserved aspartic acid and glutamine residues present within both Na^+^- and H^+^-binding sites. However, ion selectivity and stoichiometry remain unclear for most MATE transporters, with further work required to elucidate the mechanism of MATE-mediated efflux.

## SMR and PACE transporters

Transporters of the SMR family confer resistance to lipophilic compounds such as QACs and polyaromatic compounds like tetraphenylphosphonium (TPP). Composed of 100–140 amino acids arranged into four transmembrane helices, these small proteins form homo- or heterodimers to transport drugs using the PMF. As the primary representative of the family, crystal structures of *E. coli* EmrE have shown that the flexibility of its binding site is due to the rotational movements of tryptophan and glutamate sidechains, thus permitting the accommodation of various compounds. In *A. baumannii *, EmrE homologue AbeS is involved in reducing susceptibility against chloramphenicol, fluoroquinolones, erythromycin and novobiocin. It is also associated with resistance against dyes and detergents.

Although structurally similar to the SMR family with four transmembrane helices, PACE transporters are small proteins consisting of approximately 150 amino acids which confer resistance to a range of biocides and pesticides. Indeed, the founding member AceI of *A. baumannii* actively exports the broad-spectrum antiseptic agent chlorhexidine, with subsequent studies of AceI homologues further expanding the PACE substrate repertoire to include synthetic biocides such as acriflavine and proflavine, as well as short-chain diamines such as cadaverine and putrescine. As a novel family, the molecular mechanism underlying PACE-mediated extrusion remains elusive due to the lack of high-resolution structures.

## RND transporters

Members of the RND family are the most clinically significant pumps in Gram-negative bacteria because they can transport a wide range of antibiotics and are overexpressed in clinical isolates, often leading to MDR phenotypes. Each pump is a trimer with each monomer consisting of 12–14 transmembrane helices. Embedded within the inner membrane, RND pumps associate with PAPs and an OMF. Together, they form large tripartite structures that span across the entire Gram-negative cell envelope, directly extruding substrates into the extracellular space.

Using the proton motive force (PMF), RND pumps can transport a broad range of substrates with lipophilic properties (i.e. fluoroquinolones and macrolides), as well as those with cationic (antimicrobial peptides), neutral (chloramphenicol), or acidic (β-lactams) features. This poly-specificity is attributable to the flexibility of its binding pockets, which contain a hydrophobic trap predominantly clustered with phenylalanine residues, to capture non-polar ligands. The presence of polar and charged residues also create versatile multifunctional sites that allow for the capture of high-molecular-mass substrates. The majority of structure, function and assembly data for RND systems are based upon AcrAB–TolC of *E. coli *, although work on the *Pseudomonas aeruginosa* MexAB–OprM and *N. gonorrhoeae* MtrCDE systems have also contributed to our understanding.

AcrB is constitutively expressed in *E. coli *, serving as the primary RND efflux pump against antibiotics, bile salts, fatty acids and detergents to promote bacterial growth and survival. Indeed, in *Salmonella enterica* serovar Typhimurium, AcrB overexpression was induced in response to the presence of indole, cholic acid and chenodeoxycholic acid – components commonly found within the intestinal tract. Whereas many Gram-negative bacteria typically possess multiple RND systems, MtrD is the sole RND transporter in *N. gonorrhoeae *, contributing to gonococcal survival by defending against host immune cells through the expulsion of neutrophil-derived antimicrobial peptides.

RND efflux systems have also been implicated in quorum sensing (QS) and biofilm formation. QS is cell–cell communication via signalling molecules known as autoinducers (AIs), enabling bacterial cells to collectively regulate the density of a population. In *P. aeruginosa *, QS is essential for the establishment of biofilms, and MexB has been suggested to export acyl-homoserine lactones (AHLs), a type of AI commonly used by Gram-negative bacteria. Biofilms promote the colonization and long-term persistence of *P. aeruginosa *, and are often observed in chronically infected cystic fibrosis patients.

Through their overexpression and activity, multidrug efflux pumps of the RND family contribute to the survival, growth and overall virulence of Gram-negative bacteria.

## Mutations leading to multidrug resistance

While basal expression levels contribute to intrinsic resistance, increased expression of efflux pump genes promote MDR phenotypes. Indeed, *norA* overexpression has been observed in 43 % of *S. aureus* strains – particularly in MRSA strains. Likewise, overexpression of the AdeABC system is associated with MDR phenotypes of *A. baumanii* clinical isolates.

There are various regulatory mechanisms that control the gene expression of efflux pumps. At the transcription level, modulation is in response to the interactions between regulatory proteins and a number of environmental signals – many of which are currently unknown. For example, cadaverine promotes *acrAB* upregulation by binding to AcrR, causing the repressor to dissociate from the operon. Conversely, activator AceR induces PACE pump AceI expression in *A. baumannii* when bound to chlorhexidine. In *S*. Typhimurium, the two-component system (TCS) BaeSR initiates the expression of AcrD and MdtABC RND pumps to promote copper and zinc resistance.

In addition to mutations within the promoter region of efflux pump genes, the elevated expression of these transporter proteins can also result from the breakdown of its regulatory system. For instance, resistance to ciprofloxacin and QACs in *Listeria monocytogenes* has been attributed to the overexpression of MATE transporter FepA due to mutations in the repressor FepR. In comparison, upregulation of global activators results in pump overexpression – as observed with increased tigecycline resistance in *Klebsiella pneumoniae* and *Enterobacter cloacae* due to RamA-mediated overexpression of RND transporter AcrB.

Structural mutations can also alter the drugs transported by efflux pumps. For example, a G288D substitution in AcrB from a *S*. Typhimurium clinical isolate conferred resistance to ciprofloxacin, but decreased the capacity to export doxorubicin and minocycline. Likewise, *N. gonorrhoeae* clinical isolates with a K823E mutation in MtrD had decreased susceptibility to azithromycin, erythromycin and polymyxin B.

## Efflux pump inhibitors (EPIs)

Efflux pumps are potentially excellent therapeutic targets due to their considerable contribution towards antibiotic resistance and other physiological roles, including virulence and biofilm formation, in clinically relevant pathogens. Hence, EPIs are under development as potential adjuncts to antibiotic treatment.

EPIs are compounds that inhibit drug efflux to promote the intracellular accumulation of antibiotics. A prime example is phenylalanine-arginine-β-napthylamide (PAβN), a potent synthetic inhibitor of RND efflux systems. It hinders substrate uptake by competing with compounds for the RND binding pockets – although it has also been suggested to block the substrate transport. Another notable example is carbonyl cyanide m-chlorophenylhydrazone (CCCP), which prevents efflux pumps from using the PMF as an energy source. Unfortunately, although highly effective, neither compounds have therapeutic potential as both are cytotoxic even at low concentrations.

To avoid cytotoxicity, attention has shifted to screening clinically approved drugs for potential efflux inhibitor activity. For instance, the antipsychotic drug chlorpromazine inhibits AcrB efflux by binding to the transporter binding pockets. Using *S*. Typhimurium and other serovars, it was found to significantly enhance the activity of several antibiotics. Likewise, while clinically used for the treatment of various cardiac disorders, verapamil has been shown to interfere with multidrug efflux pumps by disrupting the generation of energy for efflux.

Plant-derived natural compounds have also been screened for EPI activity. For instance, *P. aeruginosa* MexXY can be inhibited by berberine, a compound extracted from plants such as goldthread and tree turmeric.

Efflux inhibitors have clear potential in the impairment of multidrug efflux pumps to restore antibiotic susceptibility. However, there are several challenges that greatly hinder their potential for medical application, such as toxicity to human cells. Ultimately, while effective in the laboratory, further work is still required before EPIs can be clinically utilized as therapeutic adjuvants to antibiotics.

## Conclusion

Multidrug efflux pumps are membrane-bound transporters involved in various cellular processes, including biofilm formation, virulence and survival. As integral components in bacterial physiology, with a clear role in the mediation of antibiotic resistance in clinically relevant pathogens, these proteins present as ideal therapeutic targets to re-establish antibiotic potency. By further understanding the structure, function and regulation of these transporters, novel therapies can be developed to overcome antibiotic resistance.

## Further reading

1. Darby EM, Trampari E, Siasat P, Gaya MS, Alav I, Webber MA, *et al*. Molecular mechanisms of antibiotic resistance revisited. *Nat Rev Microbiol*. 2022.
2. Xiao H, Zheng Y, Ma L, Tian L, Sun Q. Clinically-Relevant ABC Transporter for Anti-Cancer Drug Resistance. *Front Pharmacol*. 2021;12 : 648 407. PMC8089384
3. Robbins N, Caplan T, Cowen LE. Molecular Evolution of Antifungal Drug Resistance. *Annu Rev Microbiol*. 2017;71 : 753–75.
4. Alav I, Kobylka J, Kuth MS, Pos KM, Picard M, Blair JMA, *et al*. Structure, Assembly, and Function of Tripartite Efflux and Type 1 Secretion Systems in Gram-Negative Bacteria. *Chem Rev*. 2021;121(9):5479–596.
5. Huang L, Wu C, Gao H, Xu C, Dai M, Hao H, *et al*. Bacterial Multidrug Efflux Pumps at the Frontline of Antimicrobial Resistance: An Overview. *Antibiotics (Basel)*. 2022;11(4). PMC9032748
6. Zimmermann S, Klinger-Strobel M, Bohnert JA, Wendler S, Rödel J, Pletz MW, *et al*. Clinically Approved Drugs Inhibit the. *Front Microbiol*. 2019;10 : 2762. PMC6901667
7. Alav I, Sutton JM, Rahman KM. Role of bacterial efflux pumps in biofilm formation. *J Antimicrob Chemother*. 2018;73(8):2003–20.
8. Raturi S, Nair AV, Shinoda K, Singh H, Bai B, Murakami S, *et al*. Engineered MATE multidrug transporters reveal two functionally distinct ion-coupling pathways in NorM from Vibrio cholerae. *Commun Biol*. 2021;4(1):558. PMC8113278
9. Kornelsen V, Kumar A. Update on Multidrug Resistance Efflux Pumps in Acinetobacter spp. *Antimicrob Agents Chemother*. 2021;65(7):e0051421. PMC8218648
10. Zwama M, Nishino K. Ever-Adapting RND Efflux Pumps in Gram-Negative Multidrug-Resistant Pathogens: A Race against Time. *Antibiotics*. 2021;10(7).

